# GPCR and IR genes in *Schistosoma mansoni* miracidia

**DOI:** 10.1186/s13071-016-1837-2

**Published:** 2016-10-26

**Authors:** Di Liang, Min Zhao, Tianfang Wang, Donald P. McManus, Scott F. Cummins

**Affiliations:** 1 Faculty of Science, Health and Education, University of the Sunshine Coast, Maroochydore, QLD 4558 Australia; 2 Molecular Parasitology Laboratory, QIMR Berghofer Medical Research Institute, Brisbane, Q4006 Australia

**Keywords:** *Schistosoma mansoni*, G protein-coupled receptor, Ionotropic receptor, *Biomphalaria glabrata*

## Abstract

**Background:**

*Schistosoma* species are responsible for the disease schistosomiasis, a highly prevalent helminthic disease that requires a freshwater snail as intermediate host. The *S. mansoni* free-living miracidium must utilize olfaction to find a suitable snail host, and certain types of rhodopsin G protein-coupled receptors (GPCRs) and ionotropic receptors (IRs) have been identified as olfactory receptors in other animal phyla. The *Schistosoma* genome project, together with the recent availability of proteomic databases, allowed for studies to explore receptors within *S. mansoni*, some of which may contribute to host finding.

**Results:**

We have identified 17 rhodopsin-type GPCR sequences in *S. mansoni* belonging to four subclasses, including ligand-specific GPCRs (i.e. neuropeptide and opsin). RT-PCR demonstrated the expression of nine out of the 17 GPCRs in the free-living miracidia, each of which have been characterized for homology to *S. haematobium*. Among the nine GPCRs, two are predicted as Gq-opsins. We also describe the characterization of a *Schistosoma*-encoded IR based on similarity with other species IR and conservation of IR-like domains. *Schistosoma mansoni* IR is expressed in miracidia at 3 and 6 h post-hatch.

**Conclusions:**

The identification of receptors in *S. mansoni* miracidia, presented here, contributes not only to further understanding of *Schistosoma* biology and signal transduction but also provides a basis for approaches that may modify parasite behaviour.

**Electronic supplementary material:**

The online version of this article (doi:10.1186/s13071-016-1837-2) contains supplementary material, which is available to authorized users.

## Background

The phylum Platyhelminthes contains prominent endoparasites, including the tapeworms and flukes such as *Schistosoma* spp. [1]. Schistosomes are responsible for the disease schistosomiasis, the most prevalent and important of the parasitic platyhelminthic diseases of humans. Schistosomiasis occurs in 76 countries, affecting approximately 207 million individuals [2], and causing 280,000 deaths per year in sub-Saharan Africa [3]. This represents a serious disease burden to socioeconomic development.

During the course of its life-cycle, *Schistosoma mansoni* undergoes distinct stages of differentiation, whilst inhabiting three separate environments - freshwater, a molluscan intermediate host, and a vertebrate definitive host [4]. There are few reports that exist regarding how schistosome species interact with these environments, especially concerning the free-living miracidium stage and its infection of the intermediate snail host. Following hatching from the schistosome egg, a motile miracidium actively seeks its *Biomphalaria* host, within which it undergoes a series of developmental stages and asexual reproduction [5].

Parasites in general have evolved without many of the mechanisms needed to sustain energy for growth or reproduction without a host, for example, miracidia do not have a gut and rely solely on glycogen stored in their epidermal plate for respiration (aerobic in *S. mansoni*), and lose their infectivity once stores are depleted [6]. Therefore finding a host within a short amount of time in a potentially large body of water requires the miracidia to have highly adapted sensory mechanisms. Miracidia demonstrate host-seeking behaviors in response to chemosensory cues [1]. However, at 1–3 h post-hatch, miracidia use phototactic and geotactic cues to migrate to snail habitats, and do not respond to host chemosensory cues [7, 8]. After 1–3 h post-hatch, host attractant biomolecule(s) can be detected, reported to be non-specific small molecular weight biomolecules, and this was supported by experimental assays showing an increase in miracidia turn-back responses [9]. Further, macromolecular glycoconjugates referred to as miracidia-attracting glycoconjugates, have been implicated following an observed induction of changes to miracidial turn-back responses [10, 11]. Overall, these studies indicate that these blood flukes possess the molecular components capable of capturing, and more speculatively, processing environmental signals.

It is believed that elucidation of those molecular components that are critical for miracidial function might eventually lead to novel intervention strategies for schistosomiasis control and elimination [12]. Towards this end, recent studies have slowly unraveled insights into schistosome receptor biology and a broad range of cellular processes, such as interaction, mating and reproduction as well as the host-parasite interplay [13, 14]. G-protein coupled receptors (GPCRs) are the largest family of receptors found in eukaryotes, with more than 40 % of all pharmaceuticals targeting their various subfamilies [15]. Due to the large diversity and expansion of GPCRs between species and their ability to respond to a large selection of ligands, selectivity for GPCR-targeted anthelmintic drugs is very promising [16, 17]. GPCRs are integral membrane receptors, and respond to a multitude of extracellular ligands to transduce and amplify (or inhibit) intracellular responses involved in metabolism, neuromuscular regulation, endocrine function, vision and olfaction [15]. Key characteristics of GPCRs are 7-transmembrane spanning helical α-chains (which constitute a hydrophobic core domain), an external N-terminus and an intracellular C-terminus [18]. Among the known classes of GPCRs, the rhodopsin-type superfamily accounts for approximately 85 % of all GPCRs within many species [19] and have constituted a target of research for pharmaceuticals with many known antagonists [20–22]. Rhodopsin-type receptors are activated by a wide range of stimulants, including light, odorant molecules and neurotransmitters, and play physiological roles in vision and smell.

The availability of whole genome sequencing data has provided a basis for the *in silico* accumulation and analysis of undiscovered and potentially novel receptors in *S. mansoni* [23]. This led to the description of 117 GPCR genes belonging to five major families (105 Rhodopsin, 2 Glutamate, 3 Adhesion, 2 Secretin and 5 Frizzled) within the draft *S. mansoni* genome [17]. In 2011, the *S. mansoni* draft genome set was systematically upgraded with more than 45 % of predicted genes extensively modified and the total number reduced from 11,807 to 10,852 [24]. Employing comparative genomics, platyhelminth GPCRs have been identified and characterized for *S. mansoni* and *S. haematobium* [25]. Of those, the opsins are rhodopsin-type GPCRs that were inferred to be involved in photoreception, typically thought of as light-absorbing proteins that act as light sensors in animals [26–28]. Similar to other GPCRs, opsins have a 7-TM structure but are distinguishable from other GPCRs by a lysine residue in the seventh TM domain that binds to retinal, important for light absorption [29]. Upon light absorption, they can transform photons of light into electrochemical signals via G protein activation [30].

The first stage of eumetazoan animal chemoreception is controlled by chemosensory neurons present within the sensory epithelium, where they express olfactory receptors devoted to binding environmental odorants and transfer this information intracellularly. The accuracy of odor discrimination depends on the specificity with which odorants interact with appropriate olfactory neuronal receptors, which are often rhodopsin GPCRs [31]. The identification of rhodopsin-type GPCRs has been well studied for their role in odor detection in different animals (including humans, mouse, fruit fly, nematode and sea slug), greatly improving our understanding of the molecular mechanism of olfaction in these species [32–37]. In contrast, there is limited information on how olfaction works at the molecular level in the platyhelminths, although GPCRs have been identified as important parasite receptors with potential functions in the tegumental matrix of *S. mansoni* [38, 39].

In the animal kingdom, besides members of the superfamily of GPCRs, it has been found that ionotropic receptors (IRs) can be expressed on the olfactory sensory neurons to help confer olfactory specificity through responses to chemosensory cues [40, 41]. Unlike GPCRs, the characteristic hallmarks of IRs are their three membrane-spanning segments, a pore-forming domain and a ligand-binding Venus flytrap domain, which seems to interact with olfactory stimuli [42]. In insects, IR92a and IR76b are known to detect small amines and polyamines, respectively [43, 44].

In this study, we have used a combination of bioinformatics tools on the improved genomic database to identify *S. mansoni* GPCR genes, including opsins and putative neuropeptide GPCRs. Importantly, some are expressed in the free-living miracidium, and are possibly involved in host recognition. We also report the characterization of a schistosome-encoded IR. The identification of these receptors not only provides molecular evidence for a potential host recognition strategy in *S. mansoni,* but also contributes to the understanding of schistosome receptor biology.

## Methods

### Identification of putative GPCRs within the *S. mansoni* genome

The *S. mansoni* protein dataset used in this study was based on the improved genome assembly [24], along with expression data provided by the GeneDB (www.genedb.org) and SchistoDB (www.schistodb.net) databases. To these databases, we applied Pfam-based profile searches and identification of TM domains with the goal of identifying receptors belonging to the rhodopsin GPCR family. Specifically, this included two bioinformatic tools to predict TM domains for all proteins, including TMHMM (http://www.cbs.dtu.dk/services/TMHMM-2.0/) and Phobius (http://phobius.sbc.su.se/). As TM domains are convenient markers for GPCRs, we only focused on those sequences with 7-TM domains. Next, we applied a Pfam-based profile search using HMMerSearch (http://hmmer.org/). Proteins containing putative rhodopsin-type GPCR domains were systematically identified by profile hidden Markov model searches using the HMMer package (http://hmmer.org/) and the PFAM model PF00001 (7tm_1). Gene and protein nomenclature was based upon the *Schistosoma* gene models created from the GeneDB reference (www.genedb.org).

### Isolation of *S. mansoni* miracidia

Livers were obtained from ARC Swiss mice infected with *S. mansoni* (Puerto Rican strain), under conditions specified by the Australian Department of Agriculture, Fisheries and Forestry (DAFF). A 2-day protocol was used to obtain relatively clean schistosome eggs and miracidia [45]. In brief, the mixture of eggs and mouse liver tissue were incubated with collagenase B, penicillin and streptomycin at 37 °C overnight, followed by fractionation using Percoll columns (8 ml Percoll + 32 ml of 0.25 M sucrose in 50 ml tubes). The egg pellets were washed using PBS containing EDTA and EGTA twice on a second Percoll column (2.5 ml Percoll + 7.5 ml 0.25 M sucrose in a 15 ml tube). Purified eggs were transferred into a 200 ml hatching measuring cylinder wrapped completely in light-blocking black tape with the exclusion of the top 4 cm from the lip, thereby producing a light-gradient. The hatching cylinder was topped with pH neutral spring water until above the tape-covered area ~1.5 cm and exposed to bright light at 27 °C. Eggs were incubated for 3 h post-hatch, and the top 10 ml of miracidia-containing water (MCW) was collected for miracidia isolation; in addition, another collection was performed at 6 h post-hatch. Hatched miracidia were isolated by centrifugation at 8000× *g* for 1 min at 4 °C, and were then washed twice with water. For light microscope examination, 6 h post-hatch miracidia were fixed in 4 % paraformaldehyde on a slide, dried and washed in PBS before photographs were taken using an Olympus BX60 with Nomarski optics and a Nikon Digital Sight DS-U1 camera. For RNA isolation, miracidia were collected at 3 and 6 h post-hatch and stored separately in RNAlater.

### Reverse-transcription PCR for *S. mansoni* GPCRs

Total RNA was isolated from *S. mansoni* miracidia (3 and 6 h post-hatch) using TRIzol reagent (Invitrogen, Carlsbad, CA, USA) and RNA quantity and quality were assessed using UV spectrophotometry (NanoDrop ND-1000). First-strand cDNA was generated using random hexamer primers and the Superscript Preamplification System for First-strand Synthesis (Invitrogen). PCR was performed using primers designed (Table 1) on the CLC Genomics Workbench (v6.0; Finlandsgade, Denmark). Amplification of α-tubulin served as an internal control for the amount of RNA from each sample. Samples were heated at 94 °C for 5 min and amplified for 30 cycles (95 °C for 30 s, 45 °C for 50 s, and 72 °C for 1 min), followed by a 10 min extension at 72 °C. Reverse transcriptase negative controls were included to detect contaminating genomic DNA. The amplified DNA fragments were analyzed by 2.0 % (*w/v*) agarose gel electrophoresis.

**Table 1:** Primers used for RT-PCR of GPCR genes in *S. mansoni*

Gene ID	Forward	Reverse	bp
Smp_170020	GAGATGACTATAAGCG	CAAGTCTGGTTGTATG	303
Smp_120620	CGATGCGATAAAGATG	CTGTTAGTGTTGGATG	471
Smp_141880	TGTTAGGATGTTAGTGG	GGTTTTAGGTTGCTTG	345
Smp_104210	GGCTTGTGAGCTGTATGG	GATGTATGAAAACCTTCGGG	321
Smp_178420	ACATACCTACACCACTTCTTC	TTTGTGTTGTGCAATCGC	218
Smp_180030	ATCAATACTGGCCATGGG	CATAACGTCCAAAACCAAAG	230
Smp_041880	TGATTTTCTTGGACGTGG	GCTGGTAATAAAGCTGTTC	478
Smp_148210	TTGGTGAAGGTATGTGTG	CAGCATGTGCCATATTAC	365
Smp_043260	AAAGGCGAATTGGAAAC	CAATCACATATAGCTTCACC	387
Smp_149580	CGTTTAGTTGTATGCTGTTG	TTGGGTTGGTTGTAATGG	281
Smp_118040	GGCCTAAAGAATCATCAC	GTACATCCATAACCATCC	275
Smp_204230	CACGTAATTGGTCTGTTG	CACATATAACTGGACCAC	294
Smp_126730	AGTGAATTGTCCAGAGAAG	GTAGACCGTATTGAGCAG	358
Smp_173010	GCTGTATTTCGTGTTCTC	GTAGGATTTTGTGGATTGG	392
Smp_203500	GTAATTTGTGTTCCGTCC	TGTCGATTATCTCTTGCG	383
Smp_043320	CTCCCTCCAAATCCTATC	ATTATCCAAGCACCTCCA	371
Smp_145520	ATGATTGGAGGTGCTTGG	GTGTGTTAGAATGCTTCGG	303
tubulin	GGCGGTGGTACTGGTTCTGGG	CATTTAGCGCACCATCGAAGC	324

### Comparative analysis of *S. mansoni* and *S. haematobium* GPCRs

Multiple sequence alignments for non-opsin GPCRs were generated with Molecular Evolutionary Genetics Analysis (MEGA) software version 6.0 [46] with the MUSCLE algorithm [47]. Phylogenetic trees were constructed using the neighbor-joining method with 1000 bootstrap replicates for node support. For opsin GPCRs, a phylogenetic tree was constructed on the MEGA 6.0 platform. ClustalW [48] was used to align the sequences of the predicted proteins and the tree was constructed using the neighbor-joining and maximum-likelihood method, with 1000 bootstrap replicates for node support. Neighbor-joining and maximum-likelihood analysis was performed using no. of differences and Jones-Taylor-Thornton (JTT) method, respectively. Receptor schematic diagrams were prepared using the HMMTOP server version 2.0 (http://www.enzim.hu/hmmtop/html/document.html) [49] and LaTEX TEXtopo package [50].

### Identification and reverse-transcription PCR (RT-PCR) of *S. mansoni* IR

The *Drosophila melanogaster* IR25a [42] was used for sequence similarity searches using the NCBI tBLASTx search tool, limited to bilateria and the nucleotide dataset, resulting in identification of an EST encoding a potential IR protein within *S. mansoni*. This protein was loaded into the Pfam database (https://www.ebi.ac.uk/Tools/hmmer/search/phmmer and http://pfam.xfam.org/search), which revealed conserved ligand-gated ion channel structure. The presence of recurrent TM domain motifs was searched by TMHMM Server v2.0 (http://www.cbs.dtu.dk/services/TMHMM/). Multiple sequence alignments were performed using the MUSCLE algorithm [47] with the MEGA software (version 5.1) [46], and the LaTEX TEXtopo package [50] was used to generate schematics showing amino acid conservation.

Isolation of total RNA from *S. mansoni* miracidia followed by the two-step RT-PCR was performed in a similar manner as describe above for the GPCRs. Primers were designed for *S. mansoni IR* (sense, 5′-AGT AGA ATG CGT GAA TGG-3′ and antisense, 5′-GTT GCG GTG GTA GTC TTG-3′). Samples were heated at 94 °C for 5 min and amplified for 30 cycles (95 °C for 50 s, 46 °C for 90 s, and 72 °C for 60 s), followed by a 10 min extension at 72 °C. PCR products were visualized by 2.0 % agarose gel electrophoresis to confirm transcript expression.

### Molecular dynamics simulation for *S. mansoni* IR

The initial conformations of the receptors were built using SWISS-MODEL by sequence alignment of proteins with known 3D structures (template proteins) [51]. The structure with the highest quality estimation, based onQMEAN score, was chosen and subjected to molecular dynamics simulation (MDS) using AMBER version 14. The structure was imported using the LEAP module of AMBER; the sequence segment(s) that was missrepresented (normally at the N- or C- terminus), due to different sequence lengths of the template proteins, was built as a linear structure using LEAP and linked back to the corresponding positions. MDS was fully unrestrained and carried out in the canonical ensemble using the SANDER module. The ff14SB force field [52] was employed. Energy minimisation with 2500 steps was first performed to remove unfavourable contacts. The AMBER structure was then heated to 325 K over 50 ps to avoid being kinetically trapped in local minima [53], then subjected to unrestrained MD simulations at 325 K for the purpose of peptide equilibration. The structural information was sampled every 1 ps (i.e. 10,000 structures were calculated for 10 ns MD simulation). This MD simulation was continued until the root mean square deviation of structures within a reasonable long time range was stable at/less than 3 ~ 4 Å. Then a lowest energy structure was determined and considered as the representative of the conformations simulated over this period. Visualization of the systems was effected using VMD software [54].

## Results

### Putative GPCRs within the *S. mansoni* genome

**Fig. 1:**
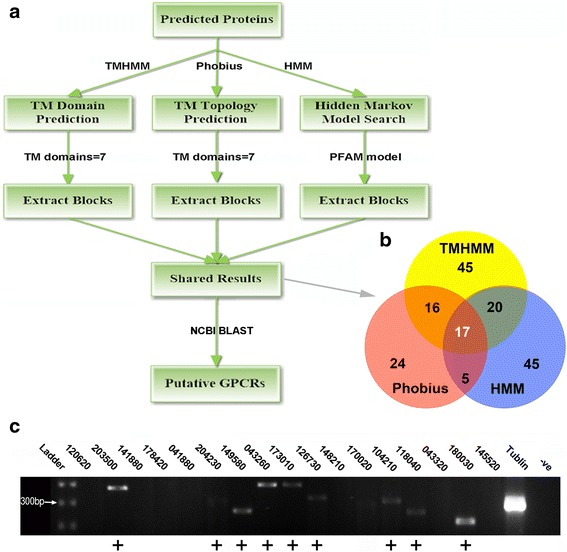
Candidate GPCRs identified from the *S. mansoni* genome. **a** Workflow for mining candidate GPCRs from the *S. mansoni* genome. **b** Venn diagram showing comparison of three bioinformatics tools (see Methods) listed to define GPCRs. **c** RT-PCR detection of *S. mansoni* miracidium GPCR transcripts at 6 h post-hatch. +, represents presence of amplicon

Using the methodology outlined in Fig. 1a, 98 proteins with 7-TM domains were extracted from the *S. mansoni* genome-derived protein models, based on TMHMM prediction. By comparison, Phobius prediction led to the identification of 62 proteins with 7-TM domains. Pfam profiling did classify 87 proteins (E-value < 0.0004) as rhodopsin-type receptors. All TMHMM, Phobius and HMMer search results can be found in the Additional file 1: Table S1.

In total, 17 genes encoding class A GPCR-like proteins (326 to 585 amino acids) were identified (Fig. 1b and Table 2) belonging to four subclasses (amine, peptide, opsin and orphan). All encode proteins considered as full-length, as determined by the presence of 7-TM domains, putative rhodopsin-type GPCR domains, as well as a methionine start and a stop codon. Table 2 also shows the amino acid identity with the identifiable homologs in the closely related *S. haematobium* [25]. Of the three new orphan GPCRs described in this paper, (i) Smp_203500 shares significant homology (95 % identity) with an allatostatin-A receptor (GenBank XP_012796783.1), (ii) Smp_204230 shares significant homology (87 % identity) with a *S. haematobium* hypothetical protein (GenBank XP_012798047.1), and (iii) Smp_178420 also shares significant homology (85 % identity) with a *S. haematobium* hypothetical protein (Genbank XP_012791982.1). RT-PCR of *S. mansoni* miracidia, using pooled samples obtained at 3 and 6 h post-hatch, revealed expression of 9 out of the 17 GPCRs (Fig. 1c). *Schistosoma mansoni* α-tubulin was used as a positive control for the cDNA templates.

**Table 2:** NCBI blast match and identity with the GPCRs for *S. mansoni*

Protein name	Aligned to *S. haematobium*	Overall identity (%)	GPCR subclass [17]	NCBI BLAST best hit (Reference/Name/Species)	Identity (%)
Smp_043320	Sha_102744	75	amine	emb|CCD79388.1| Tyramine-like receptor [*S. mansoni*]	100
Smp_126730	Sha_Exo_8	94	amine	emb|CCD75608.1| 5-HT-like receptor [*S. mansoni*]	100
Smp_145520	Sha_104648	53	amine	emb| CCD79384.1| Putative adrenoreceptor [*S. mansoni*]	100
Smp_148210	Sha_101211	33	amine	emb|CCD77402.1| 5-HT-like receptor [*S. mansoni*]	100
Smp_043260	Sha_Exo_1	87	amine	emb|CCD79382.1| Putative GPCR receptor [*S. mansoni*]	100
Smp_141880	Sha_101834	76	peptide	emb|CCD81490.1| Neuropeptide-like receptor [*S. mansoni*]	100
Smp_118040	Sha_108334	86	peptide	emb|CCD75717.1| Neuropeptide-like receptor [*S. mansoni*]	100
Smp_041880	Sha_104441	88	peptide	emb|CCD79664.1| Peptide-like receptor [*S. mansoni*]	100
Smp_149580	Sha_101834	80	peptide	emb|CCD59781.1| FMRFamide-like receptor [*S. mansoni*]	100
Smp_170020	Sha_104691	86	peptide	emb|CCD82074.1| Neuropeptide-like receptor [*S. mansoni*]	100
Smp_120620	Sha_102107	19	peptide	emb|CCD59108.1| Amine GPCR-like receptor [*S. mansoni*]	100
Smp_104210	Sha_101185	80	opsin	gb|AAF73286.1| Opsin-like receptor [*S. mansoni*]	100
Smp_180030	Sha_101097	85	opsin	emb|CCD82678.1| Opsin-like receptor [*S. mansoni*]	100
Smp_173010	Sha_107429	66	orphan	emb|CCD76373.1| Myosuppressin-like receptor [*S. mansoni*]	100
Smp_204230	Sha_105544	87	orphan	ref|XP_012798047.1| Hypothetical protein [*S. haematobium*]	87
Smp_178420	Sha_108078	85	orphan	ref|XP_012791982.1| Hypothetical protein [*S. haematobium*]	85
Smp_203500	Sha_105605	95	orphan	ref|XP012796783.1| Allatostatin receptor [*S. haematobium*]	95

### Comparative analysis of GPCRs present in *S. mansoni* miracidia with *S. haematobium*

**Fig. 2:**
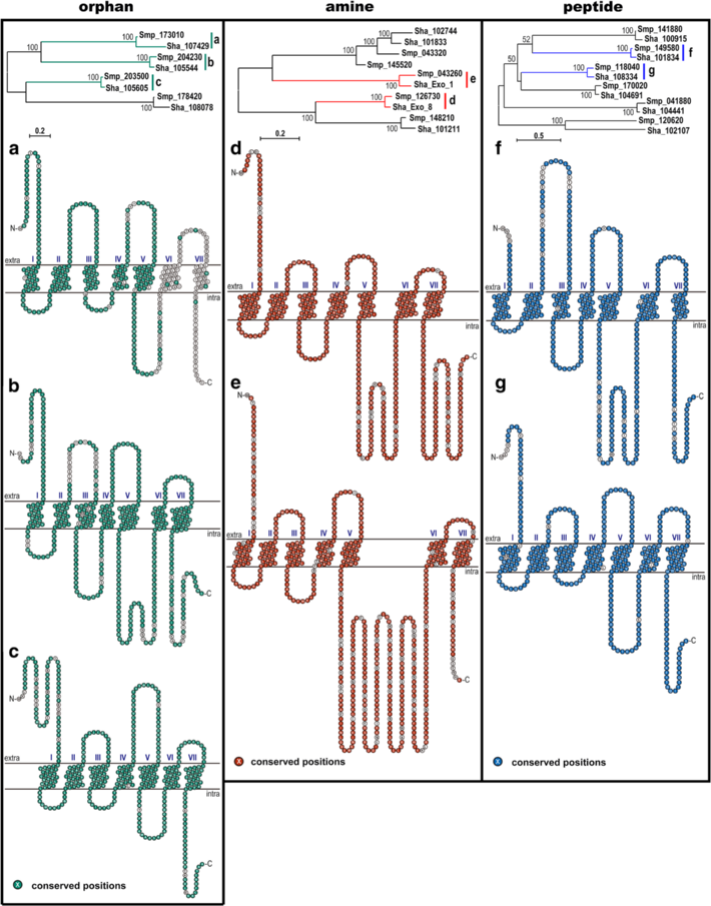
Comparative analysis of *S. mansoni* and *S. haematobium* orphan, amine and peptide GPCRs. According to their corresponding GPCR subclasses, phylogenetic trees were constructed using candidate orphan (**a-c**), amine (**e-d**) and peptide (**f-g**) GPCRs identified from the *S. mansoni* genome compared with the *S. haematobium* homologs. Schematic representation of GPCR sequences identified in *S. mansoni* miracidia along with the associated the *S. haematobium* homologs, showing conserved amino acids. Smp and Sha represent the GPCR sequences from *S. mansoni* and *S. haematobium*, respectively

According to their corresponding sub-classification, phylogenetic trees were constructed for each subclass using the final set of predicted non-opsin GPCRs grouped with the *S. haematobium* homologs (Fig. 2), confirming the high phylogenetic similarity. Schematic GPCR representations show specific regions of conservation and divergence between species homologs. Most sequence divergence was noted between the orphan receptors Smp_173010 and Sha_107429, specifically within the region containing the TM6 domain through the C-terminus. The amine-type GPCRs showed most variability within the intracellular loop 3 region. Overall, there was very high conservation observed within the peptide-type GPCRs, although some variability is present within the N-termini region.

**Fig. 3:**
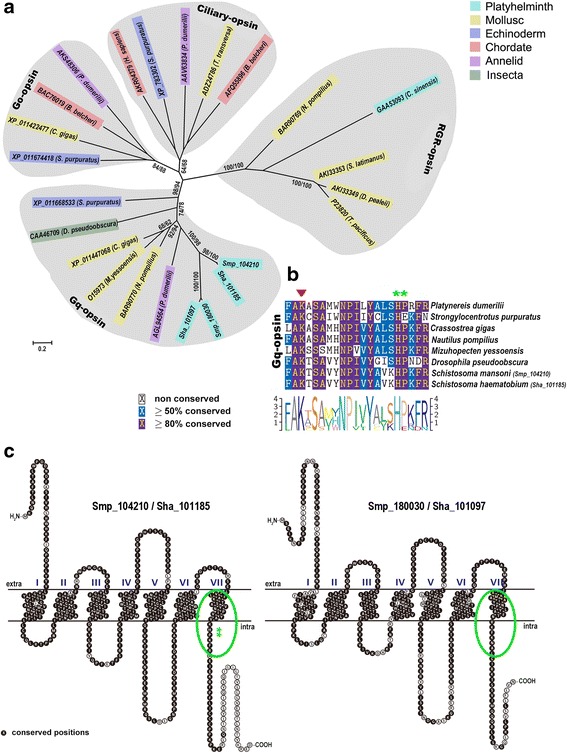
Phylogenetic analysis and characterization of opsins. **a** Phylogeny showing that representative bilaterian opsin members cluster into four supported subfamilies. Smp, *S. mansoni* and Sha, *S. haematobium*. Branch support values are indicated next to branching points. Maximum-likelihood bootstrap support values (significant support threshold value >60 %) are indicated as the first set of numbers at the nodes. Neighbor-joining bootstrap support values are indicated as the second set of numbers at the nodes, with bootstrap value expressed values above 60 % shown. Scale-bar indicates the relative amount of amino acid changes. **b** Partial amino acid alignment of members of the Gq-opsin subfamilies. *Asterisks* demarcate the fingerprint indicative of Gq-opsin families. An *arrowhead* demarcates the position of the lysine residue critical for Schiff base formation. **c** Schematic representation of *S. mansoni* opsin compared with the *S. haematobium* homologs. *Circle* shows region from panel **b** and *asterisks* demarcate presence of the Gq-opsin fingerprint

Amongst the *S. mansoni* rhodopsin-type GPCR genes, two sequences (*Smp_104210* and *Smp_180030*) were predicted as opsin GPCRs. These encode opsin-like receptors that share the greatest degree of conservation to two *S. haematobium* sequences (Sha_101185 and Sha_101097) (Table 2). We confirmed the clustering of four distinct ancient bilaterian opsin subfamilies (Fig. 3a), namely the Gq-opsins, ciliary opsins, Go-opsins as well as members of the retinal-photoisomerase subfamily, which includes retinal GPCR (RGR) and retinochrome. As might be expected, Smp_104210 and Smp_180030 grouped with Sha_101185 and Sha_101097 in a pairwise, orthologous manner within the Gq-opsin group. Partial sequence alignment of members of the Gq-opsin subfamilies, specifically within the cytosolic region of the TM 7 domain and C-terminal tail, demonstrates two highly conserved peptide amino acids [Histidine, Proline (H, P)] in the carboxy terminal intra-cellular loop domain that are highly indicative of Gq-opsin families (Fig. 3b, c). This distinctive characteristic is conserved in both *S. mansoni* Smp_104210 and *S. haematobium* Sha_101185. No such motif was detected for the other opsin-like GPCR (Smp_180030 and Sha_101097).

### Identification of IRs within *S. mansoni*

A single *S. mansoni IR* was identified from the genome that encodes a conventional ligand-gated ion channel domain protein (513 aa; 58.7 kDa). This receptor, *S. mansoni* IR, displays remnants of classical IR motifs at corresponding positions and predicted domains that are critical structural regions responsible for detecting odor ligands and contributing to ligand specificity, including an extracellular two-lobed ligand-binding domain and four features common to all conventional IRs, namely: (i) IR-related motifs with TM stretches, (ii) possession of Pfam domains PF10613 and PF00060, which are specific for the ligand-gated ion channel receptors, (iii) highly-conserved structural features specifically shared amongst the IR family, and (iv) a region surrounding the ligand-binding domain. All are present within the *S. mansoni* IR receptor showing considerable conservation with the IRs of other species, including the well-studied *D. melanogaster*.

**Fig. 4:**
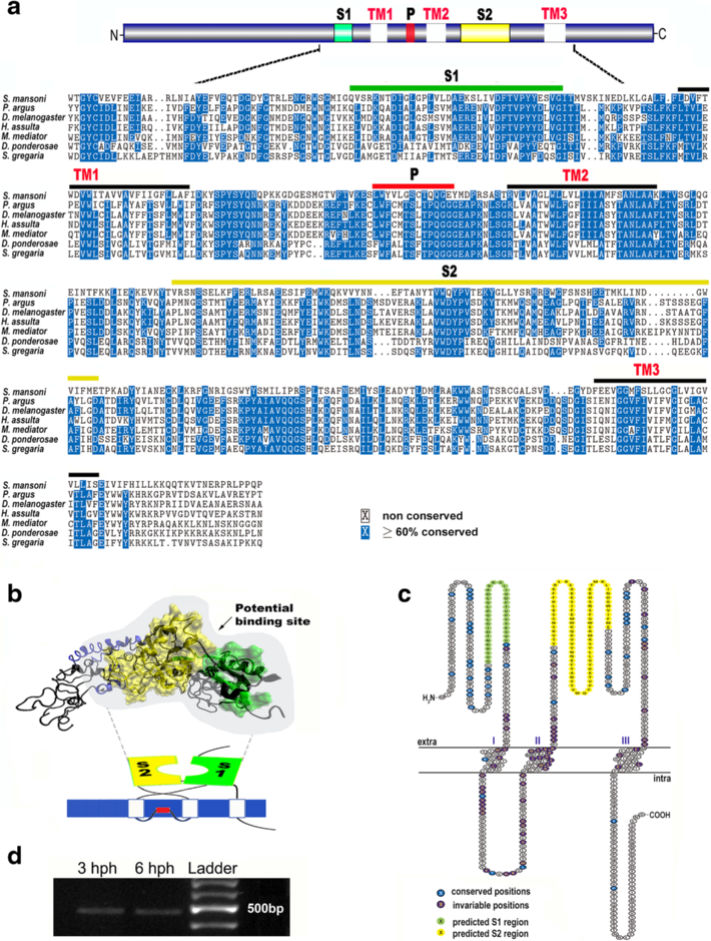
Characterisation of *S. mansoni* IR. **a** Sequence analysis of *Schistosoma* IR and in comparison with IR from other species. *Top*: The predicted protein domain organization of typical IR is shown in histogram. *Below*: Alignment of *Schistosoma* IR with IR proteins of representative selections from other species. Domains: TM, transmembrane domain; S, ligand-binding domain; P, pore. **b** Structure of *S. mansoni* IR as predicted by MDS model with space filling of predicted binding sites. *Yellow* shows the predicted ligand binding S1 region, *green* shows the predicted ligand binding S2 region, and *blue* demarcates the predicted TM regions. The protein domain structure of conventional IRs in cartoon form is shown below [40, 41]. **c** Schematic representation of all IRs shown in **a**, demonstrating conserved and invariable amino acids, as well as predicted S1 and S2 regions. **d** RT-PCR showing expression of *IR* in *S. mansoni* miracidia at 3 h and 6 h post-hatch (hph)

A representation of the *S. mansoni* IR compared to six Protostomia species, including *Panulirus argus*, *Helicoverpa assulta*, *Microplitis mediator*, *Dendroctonus ponderosae*, *Schistocerca gregaria* and *Drosophila melanogaster* was used to unify protein structure predictions across species (Fig. 4a). Sequences used for this analysis are provided in Additional file 2: Table S2. All IRs display classical IR motifs at corresponding positions and critical structural regions responsible for binding ligands and contributing to ligand specificity. Figure 4b, c demonstrates the proposed structure model of the Venus flytrap domain of the schistosome IR, and with the putative ligand binding sites. RT-PCR results demonstrate *S. mansoni IR* expression within the free-living miracidia at both 3 and 6 h post-hatch (Fig. 4d).

## Discussion

Schistosome miracidia must find an appropriate host within a very limited time-span, thus it would seem advantageous for them to have evolved finely-tuned molecular strategies allowing for host detection, thereby increasing the likelihood of successful snail infection. GPCRs and IRs are fundamental to chemoreception in many animal species [55–57], and we speculate this may also be similar for the *Schistosoma* miracidia. In this study, we have analyzed the *S. mansoni* genome to identify putative receptors, and specifically those present within the miracidium, some of which may be important for snail host-finding.

We identified 17 rhodopsin-type GPCRs that belong to amine, peptide, opsin and orphan groups. Among these, the amine group consisted of biogenic amine receptors such as serotonin, dopamine and histamine, that have a prominent role in the flatworm nervous system [58, 59]. Of these, experimental validation has been established for the histamine receptor (Smp_043260) [60]. Regarding the other GPCRs identified in this study, three (Smp_203500,Smp_204230 and Smp_178420) have not been described previously, while Smp_173010 was reported by Campos et al. [25] as novel platyhelminth-specific rhodopsin-like orphan family (PROF). This variation can be explained by the different workflow for curation of the final GPCR list, whereby receptors were only taken if they satisfied requirements within all of TMHMM, HMM and Phobius tools. Smp_203500 and PROF receptors Smp_173010 do have some similarity to characterized receptor types, allatostatin and myosuppressin, respectively; however this has not been experimentally validated. Platyhelminthes appear to lack a conventional endocrine system [38], and are therefore heavily reliant on neural signalling via neuropeptides that control vital dynamic physiological and neural functions, such as growth, reproduction, host-seeking olfaction, locomotion, immune evasion and sexual dimorphism [61]. The importance of their peptidergic neural and associated receptor systems holds a promising area of research for new anthelmintic drug targets [16, 62, 63].

RT-PCR demonstrated the expression of nine out of the 17 GPCRs in the free-living miracidia, suggesting that these receptors are possibly either involved in miracidia host-finding and recognition or required for miracidia metabolism, including the histamine receptor. Representative comparative schematic models demonstrated the divergence in amino acid sequence of these GPCRs and homologs in *S. haematobium*, suggesting the potential biological differences between these two schistosomes. Notably, PROF receptor Smp_173010 shows relatively large variation in protein sequence within the C-terminal region, thus we speculate that there may be binding of species-specific ligands through this receptor.

The opsin-like GPCR Smp_104210 has been reported as being differentially regulated during the parasite’s life-cycle and, in the cercaria, it localizes to organelles found directly below the parasite’s epidermis, associated with organelles within the vicinity of the most anterior osmoregulatory flame cells [64]. Based on this morphological description, it likely acts as a photoreceptor responsible for the direct photokinetic behavior of cercaria in response to light [64]. Further, annotation of opsin-like GPCR Smp_180030 has been determined as a result of the analysis of RNA-seq expression profiles [24], yet no role in schistosome photoreception processes has been reported.

As indicated by our phylogenetic analysis, Smp_104210 and Smp_180030 have well-supported clustering with other Gq-opsin genes involved in photosensation that strongly implies that these receptors are Gq-opsins. The detection of both receptors (Smp_104210 and Smp_180030) in light-responsive miracidia, as well as their clustering with other Gq opsins, suggests that they may serve an integral role in host-finding by participating in *Schistosoma* photokinesis [8, 64–66].

We identified a single *S. mansoni* IR. The IR repertoire throughout protostomes shows substantial variation in size, ranging from three in *C. elegans* to 85 in the crustacean *Daphnia pulex* [67, 68]. The *S. mansoni* IR exhibits the typical venus flytrap structure (see Fig. 4b) and shares sequence similarity to characterised IRs of other species. Another type of venus flytrap receptor has been studied in *S. mansomi*, known as the venus kinase receptor [69]. There are two isoforms of this receptor, one that binds L-arginine (SmVKR1) and another that binds calcium ions (SmVKR2), which are thought to be important for development and reproduction. Like the IR identified in our study, the ligand for the *S. mansoni* venus kinase receptor is unknown. IRs in the insects are known to bind polyamines [43], yet may also act as thermosensors [70]. We speculate that the *S. mansoni* IR could play an important role as a chemosensory and/or thermosensory receptor in different life-cycle stages, supported by its observed expression profile in cercariae, schistosomula and adults (GeneDB, version 4.0).

## Conclusions

The characterization of GPCRs and IRs in *S. mansoni* is likely to inform us about their pharmacological profiles and features towards manipulating chemosensory-driven behaviors. Given that *S. mansoni* IR and at least some of the GPCRs are expressed in the miracidium, we hypothesize they may be dedicated to detect specific odor cues, including responses to odors emitted from *Biomphalaria*. As these odors are likely instrumental for parasite transmission, GPCRs and IRs may represent proteins against which novel prophylactic therapies can be developed.

## Additional files

Additional file 1: Table S1. TMHMM, Phobius and HMMer search results during the identification of putative GPCRs within the *S. mansoni* genome. (XLSX 98 kb)
Additional file 2: Table S2. Sequences used for phylogenetic analysis in Fig. 4a. (XLSX 19 kb)

## Abbreviations

GPCRs: G protein-coupled receptors
HMM: Hidden Markov Model
IRs: Ionotropic receptors
MDS: Molecular dynamics simulation
MEGA: Molecular evolutionary genetics analysis
PROF: Platyhelminth-specific rhodopsin-like orphan family
RGR: Retinal GPCR
TM: Transmembrane
